# Unconventional Secretion of Plant Extracellular Vesicles and Their Benefits to Human Health: A Mini Review

**DOI:** 10.3389/fcell.2022.883841

**Published:** 2022-06-01

**Authors:** Joshua T. Farley, Mahmoud K. Eldahshoury, Carine de Marcos Lousa

**Affiliations:** ^1^ Biomedical Sciences, School of Health, Leeds Beckett University, Leeds, United Kingdom; ^2^ Centre for Plant Sciences, University of Leeds, Leeds, United Kingdom

**Keywords:** extracellular vesicles, unconventional protein secretion (UPS), plant EVs, biomedicine, biopharming, exosomes

## Abstract

Mechanisms devoted to the secretion of proteins via extracellular vesicles (EVs) have been found in mammals, yeasts, and plants. Since they transport a number of leader-less proteins to the plasma membrane or the extracellular space, EVs are considered part of Unconventional protein secretion (UPS) routes. UPS involving EVs are a relatively new field in plants. Aside from their role in plant physiology and immunity, plant extracts containing EVs have also been shown to be beneficial for human health. Therefore, exploring the use of plant EVs in biomedicine and their potential as drug delivery tools is an exciting avenue. Here we give a summary of the state of knowledge on plant EVs, their crosstalk with mammalian systems and potential research routes that could lead to practical applications in therapeutic drug delivery.

## 1 Introduction

Extracellular vesicles (EVs) are a collection of vesicles with different origins, size ranges, and molecular composition. Originally considered as cellular waste, their discovery has revolutionised our understanding of cell-cell communications and transfer of biological information from 1 cell to another. Since leaderless proteins loaded in these vesicles bypass the Golgi and are recruited in EVs from the cytosol, most EVs are considered part of the unconventional secretion pathway (UPS). Exosomes, a particular type of EV, are particularly interesting in this context for the following reasons: the mechanism of cargo loading *in vivo* and *in vitro* are being better understood in human cells (Xu et al., 2020), exosomes have the ability to cross natural barriers (Blood brain barrier and placenta) and are described as safe and stable nanoparticles (Banks et al., 2020; Elliott and He, 2021). Consequently, mammalian exosomes are being investigated for their potential in drug delivery (Xu et al., 2020; Choi et al., 2021). Plants also secrete extracellular vesicles, and exosomes have been identified (He et al., 2021). While keeping the benefits of human exosomes, the use of plant exosomes as drug delivery tools in biomedicine might offer various additional advantages such as lower production costs involved in biopharming and reduced cross-human contaminations. In this mini-review, we are summarising the current knowledge on plant UPS specifically focusing on EVs and exosomes. We are then clarifying the extraction procedures of various plant EVs and finally we are proposing a view on the potential benefits of using plant EVs as drug delivery tools in human health.

### 2 Linking UPS and EVs in Mammals and Plants

#### 2.1 Mammalian UPS and EVs

Unconventional protein secretion (UPS) involves a range of mechanisms that allow proteins to reach the extracellular medium, bypassing at least part of the conventional ER-Golgi-PM secretory pathway. While this conventional pathway usually involves the presence of signal peptides at the N-terminus of proteins, UPS leads to the secretion of leaderless soluble proteins in the extracellular medium or trafficking of membrane proteins via an alternative route than through the Golgi (Rabouille et al., 2012; Rabouille, 2017). These mechanisms are being intensively studied in mammals and yeasts because they are often associated with stress and pathologies such as inflammatory diseases or cancer (Kim et al., 2018; Cohen et al., 2020). Therefore, understanding the mechanisms of UPS is a promising new route into identifying new therapeutic targets. Extracellular vesicles, in particular, represent a specific type of vesicular UPS that has been extensively studied since their discovery 40 years ago (Harding et al., 2013). Their ability to pack biological information which is then transmitted to adjacent or long-distance cells have triggered extensive research into their use as a drug delivery system. There are various types of extracellular vesicles that can be classified depending on their origin and content (Théry et al., 2018). This classification is constantly updated with new knowledge. Exosomes, a specific class of small EVs (sEVs) released by the fusion of MVBs with the membrane, are of particular interest for targeted drug delivery since they have been shown to cross natural barriers such as the Blood brain barrier and placenta (for review Elliott and He, 2021). The use of mammalian exosomes in drug delivery presents various advantages described above but also some challenges (Meng et al., 2020; Chen et al., 2021). Three of these challenges are the lack of homogeneity, the lack of large-scale cost-effective production, and ethical issues linked with transferring human material.

### 2.2 Plant UPS and EVs

To address some of these challenges in terms of cost-effective production and lack of ethical issues, plants might offer an alternative source of exosomes and EVs. As a result, a growing number of studies are looking into their potential health benefits. For example, the effect of plant extracellular vesicles loaded with curcumin are currently being tested in clinical trials (NCT01294072) to evaluate their impact on surgery of newly diagnosed colon cancer patients (https://clinicaltrials.gov/ct2/show/NCT01294072).

Unfortunately, plant unconventional protein secretion pathways have attracted only late interests and our current knowledge of plant UPS and EVs is growing but still limited (Ding et al., 2014a; Robinson et al., 2016; Hansen and Nielsen 2017; Cui et al., 2019). The presence of leaderless proteins in apoplastic extracellular vesicles has confirmed that these EVs represent genuine plant UPS pathways involved in cell wall remodelling and resistance to infection (Delaunois et al., 2013, 2014). Investigations around these vesicular mechanisms have uncovered the existence of at least three pathways that result in the release of extracellular vesicles in plants: exocyst-positive organelle mediated secretion (EXPO), vesicle budding from the PM (including microvesicles), and multivesicular body (MVB)-PM fusion (Wang et al., 2010; Regente et al., 2012; Cui et al., 2019). A growing number of studies report the beneficial effect of crude and pure extracts of plant EVs on human health (Akuma et al., 2019; Alfieri et al., 2021; Urzì et al., 2021). To evaluate their potential as drug delivery tools, the current state of the field in terms of plant EVs classification, purification, and biomedical applications is presented below.

### 3 Plant EV Classification and Isolation

#### 3.1 Plant EV Subtypes and Biogenesis

**FIGURE 1:**
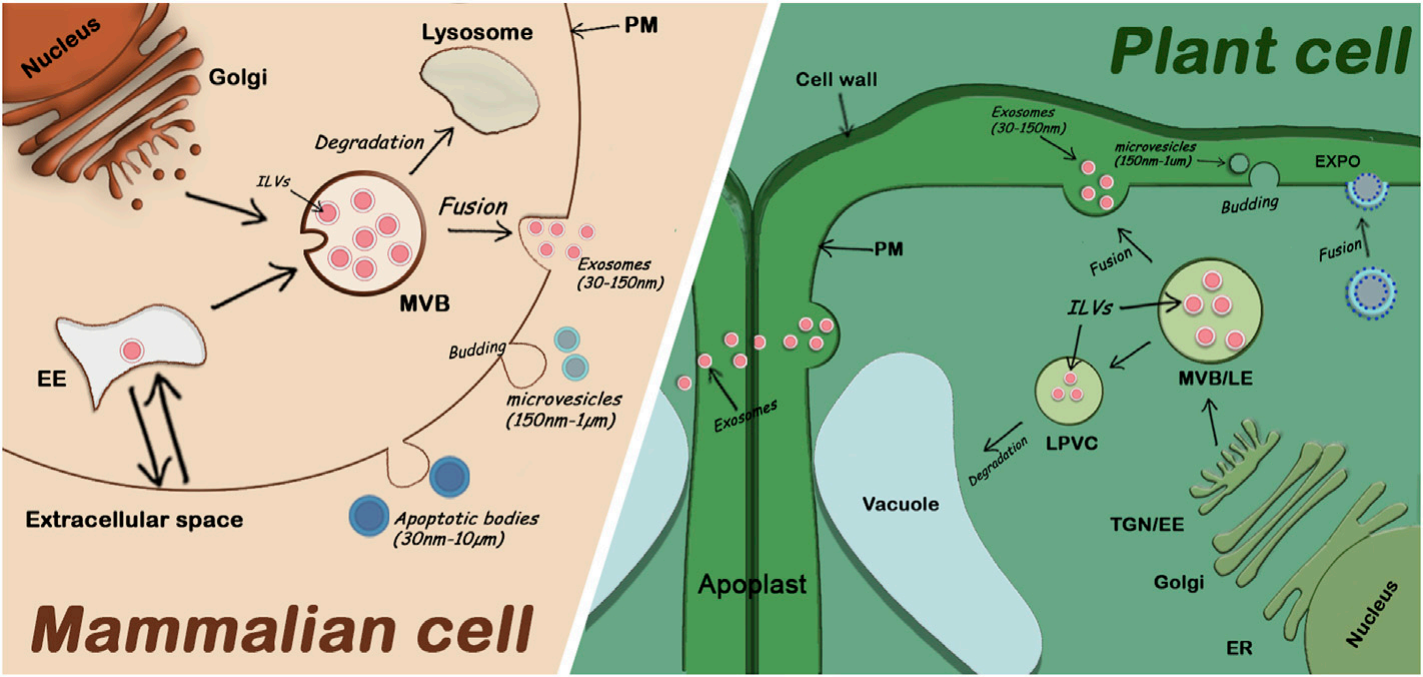
Comparison of extracellular vesicle secretion in mammalian cells and plant cells. Mammalian EVs including apoptotic bodies, microvesicles, and exosomes are secreted in the extracellular medium. Plant EVs are also secreted in the extracellular medium (the apoplast). Exosomes are secreted by fusion of MVBs with the PM, EXPO vesicles are also secreted by fusion with the PM while microvesicles and apoptotic bodies are released through budding of the PM. EE: Early Endosome; ER: Endoplasmic Reticulum; ILVs: Intraluminal Vesicles; LE: Late Endosome; LPVC: Late Pre-vacuolar Compartment; MVB: Multivesicular Body; PM: Plasma Membrane; TGN: Trans Golgi Network; (proportions of organelle sizes not conserved).

The term “plant extracellular vesicles” generally refers to apoplastic vesicles. Plant-derived nanovesicles (PDNVs) or exosomes-like nanoparticles (ELNs) are terms used to refer to vesicles that have been isolated from total plant extracts and usually contain a mix of EVs and other cellular microvesicles (Pinedo et al., 2021). Since the identification of specific markers for different EV subclasses is only recent, the classification of plant EVs is not well established, but three main classes have been described (Cai et al., 2021). One class involves EXPO vesicles secreted into the apoplast after the fusion of EXPO double membrane organelles with the plasma membrane. The second class includes microvesicles (or ectosomes), suggested to be smaller (150nm-1um) and originate by budding from the plasma membrane. Finally, exosomes (30–150 nm) are the third class of plant EVs and are released by fusion of MVBs (containing intraluminal vesicle) with the plasma membrane (Figure 1). The mechanisms by which all these fusions and releases in the extracellular space occur are not well understood in plants.

While Exo70E2 protein has been identified as a marker of EXPO vesicles, it has been reported that exosomes specifically contain TET8, a tetraspanin protein (Wang et al., 2010; Cai et al., 2018). This assumption is supported by the fact that TET8 is a plant orthologue for the human exosomal marker CD63 (Théry et al., 2018). In addition, the density of TET8 fraction (1.12–1.19 g/ml) isolated at 100,000 g correlates with the density of human exosomes, and TET8 is found to colocalize with MVB markers (He et al., 2021). Microvesicles, on the other hand, appear to be positive for the syntaxin SYP121, which has often been referred to as PEN1 (Ding et al., 2014; Rutter and Innes, 2017; He et al., 2021). The SYP121/PEN1-positive fraction appears to be slightly less dense (1.029–1.056 g/ml), and contains larger vesicles ranging from 50 to 300 nm that can be pelleted at 40 000 g (Rutter and Innes, 2017). SYP121/PEN1 has also been reported to be involved in Golgi-PM trafficking, reinforcing the fact that SYP121/PEN1 positive vesicles might not be of MVB origin (Nielsen et al., 2012; He et al., 2021).

### 3.2 Plant EV Isolations for Drug Delivery

The processes described to isolate plant EVs depend on the nature of the plant material. Apoplastic fluids are usually extracted from leaves, while blending/juicing is performed on fruits or roots. EVs can also be isolated from liquid plant exudates (Araya et al., 2015). Although the purities of different EV fractions will vary, they have all been found to have therapeutic potential in biomedicine.

### 3.2.1 Apoplastic Washing

The apoplast is the space outside the plasma membrane of plant cells where material can freely move (Sattelmacher, 2001). Although it is unknown how EVs cross the cell wall, their presence in the apoplast has been confirmed (Regente et al., 2012; Rutter et al., 2017; He et al., 2021). To recover these vesicles, a standard technique based on vacuum-infiltration and ultracentrifugation is performed (O’Leary et al., 2014). Applying sequential rounds of negative and atmospheric pressure onto leaves forces a buffer into the apoplastic space that can be recovered after centrifugation of the leaf. This method ensures that plant cells remain mostly undamaged and results in a relatively pure fraction containing EVs but depleted of intracellular components. It has been mostly used to purify EVs from leaf material (*Arabidopsis thaliana*, Nicotiana benthamiana) or seeds (sunflower) (Regente et al., 2009; Rutter and Innes, 2017; Zhang et al., 2020). Additional purification steps will allow further isolation of different types of EVs as described above (Regente et al., 2009; Rutter and Innes, 2017; He et al., 2021). Recently, a comparative analysis of two major methods for isolating EVs from apoplastic wash fluids has provided a guide into the selection of the right method adapted to the type of downstream applications desired (Huang et al., 2021).

### 3.2.2 Blending or Juice Extraction

Enriched EV fractions have been obtained through blending plant matter such as ginger roots, herbs, wheat, and dandelion (Mu et al., 2014; Xiao et al., 2018; Chen et al., 2019). Juicing of citrus fruits, pears, grapefruit, watermelons, and coconut water has also been used to prepare EV extracts (Liang et al., 2015; Raimondo et al., 2015; Xiao et al., 2018; Zhao et al., 2018). However, unless they are subjected to further purification steps, these methods often result in a mix of EVs and intracellular content (vesicles, organelles, membranes), meaning they are not solely products of UPS (Pinedo et al., 2021). They are, therefore, referred to as Plant-derived nanovesicles (PDNVs) or Exosome-like nanovesicles (ELNs) rather than EVs which refer to the purer fractions. There is increasing evidence that these PDNVs have significant biological effects on human cells and have brought new hope into novel forms of natural drug delivery systems (Di Gioia et al., 2020; Alfieri et al., 2021; Urzì et al., 2021).

### 3.2.3 Plant Exudates

Plant exudates are substances excreted from plants that include liquids flowing through and out of plants. This includes sap, gum, resins or root exudates. They have been used for many years in traditional medicine. Exudates contain many bioactive compounds, amongst them peptides, with beneficial effects on human health such as reduction of oedema and inflammation (Licá et al., 2018). Plant EVs derived from exudates are a relatively new research topic. EVs isolated from the sap of two plants (namely Dendropanax morbifera, and Pinus densiflora) have shown cytotoxic and anti-metastatic effects on human tumour cells (Kim et al., 2020a; Kim et al., 2020b). Furthermore, EVs from a hydroponic solution containing tomato (Solanum lycopersicum L.) root exudates were shown to inhibit the spore germination of three fungal phytopathogens (*Fusarium* oxysporum, Botrytis cinerea and *Alternaria alternata*) suggesting an antifungal activity in plants (De Palma et al., 2020). Whether this activity can be applied to mammalian fungal pathogens has not been tested. More research is needed to understand if exudates EVs could hold promising therapeutic applications.

### 4 Plant EVs as a Drug Delivery Tool

Plants have been known for centuries to be beneficial for human health. Yet the identification of extracellular vesicles and their molecular content shed a new light on our understanding of cross-kingdom interaction and transfer of bioactive molecules.

### 4.1 Benefits of Plant PDNVs Bioactive Compounds

In the past decade, numerous reports have described the beneficial effects of plant PDNVs/EVs in mammalian health. While PDNV proteomes from various plant origins have been characterised and some common proteins frequently identified in these vesicles, the variety of PDNVs and the lack of specific protein markers limits their classification which may prove problematic for large scale good manufacturing practices (GMPs). Nevertheless, PDNVs contain a range of bioactive molecules such as proteins, lipids, or metabolites with therapeutic effects summarised in (Woith et al., 2019; Di Gioia et al., 2020; Kocak et al., 2020; Alfieri et al., 2021; Urzì et al., 2021). Amongst the most studied plant PDNVs are those originating from ginger. These EV-containing PDNV isolates have many natural therapeutic potentials and can induce physiological changes in mammals. They were shown to influence the human gut microbiota (Teng et al., 2018), inhibit inflammasome activation (Chen, Zhou and Yu, 2019), and found to have a positive effect on inflammatory bowel disease and colitis-associated cancer (Zhang et al., 2016). They have also been shown to be taken up by, and inhibit the pathogenicity of, the periodontitis-causing Porphyromonas gingivalis (Sundaram et al., 2019). In parallel, wheat derived nanovesicles have been shown to aid *in vitro* wound healing by promoting proliferation and migration of dermal fibroblasts, endothelial, and epithelial cells (Şahin et al., 2018). Nanovesicles derived from various fruits and vegetables were also shown to inhibit cancer cell growth (Kameli et al., 2021). Despite their numerous health benefits, it is unclear however, if this positive impact is attributable to the combined action of various bioactive components in the crude fraction or to particular compounds that may be isolated from purer EVs preparations.

### 4.2 Plant EV Engineering and Biopharming

Research on EVs (obtained from the apoplast of plants) as potential drug delivery systems is far more restricted than those on PDNVs. So far, to our knowledge, only one study has shown that purified apoplastic small EVs (sEVs) are efficiently taken up by human ovarian cancer cells OVAR5 (Liu et al., 2020). This paper compared the uptake of apoplastic sEVs (purified from the apoplast of Arabidopsis leaves) and nanovesicles (obtained from disrupted leaf material). OVAR5 cells were found to be significantly more susceptible to apoplastic sEV uptake than leaf nanovesicle uptake, based on elevated numbers of fluorescent cells. These results suggest that pure EV samples have the same, if not greater, drug delivery potentials than PDNV isolates have, and that EVs may be the contributing factor to PDNV success. Unfortunately, to our knowledge, this is the only study that uses purified apoplastic EVs in human cells and more data is required to conclude. In addition, an assessment of immunogenicity and toxicity should be undertaken to validate pure plant EVs as a drug delivery system.

Based on the successes of PDNVs, efficient uptake of sEVs, and the potential of engineering exosomes in plants, biopharming is an attractive solution to produce cheap pharmaceuticals with a rapid turnover. Biopharming, or plant molecular farming, refers to the use of genetic tools to produce a wide range of pharmaceuticals. Plants have already been used to produce antibodies and vaccines for humans, animals, and aquaculture (Shoji et al., 2012; Takeyama et al., 2015; Yao et al., 2015; Lefebvre and Lécuyer, 2017; Zahara et al., 2017; Su et al., 2021). Recently, plants have been explored as a rapid alternative biofactory for the production of COVID vaccines through the expression of Virus-like particles exposing an immunogenic part of the Spike S protein (Dhama et al., 2020; Maharjan and Choe, 2021). Regarding clinical trials, intravenous administration of *β*-glucocerebrosidase protein expressed in carrots has been approved as being safe and efficient and successfully used for 2 decades (Shaaltiel et al., 2015). The advantages of using plants as Biofactories include their ability to produce functional proteins in large amounts, and at lower costs (Shaaltiel et al., 2007). One additional advantage is the possibility of relatively simple engineering associated with plants, potentially allowing *in vivo* packaging of exogenous cargo into EVs, ready for extraction. More data on the mechanisms of loading into plant EVs is still required, but with this possibility in mind, and given that delivery of therapeutic molecules by mammalian EVs has already been demonstrated by several studies (Alvarez-Erviti et al., 2011; Batrakova and Kim, 2016; Elsharkasy et al., 2020), biopharming plants to isolate therapeutic pure EVs is a very exciting avenue that needs to be explored.

### 4.3 Administration and Bioavailability

If plant EVs are to be potential drug delivery systems, their administration and bioavailability must be considered. The first strong evidence of cross kingdom effects was provided when isolated PDNVs were fed to mice and found to reach intestinal macrophages. The vesicle uptake in these cells increased the expression of interleukins and alleviated colitis symptoms (Ju et al., 2013; Mu et al., 2014). This study has demonstrated that PDNVs are able to resist gastric and intestinal digestion, suggesting oral administration methods of plant nanoparticles are suitable for targeting these organs. In order to reach other organs, alternative administration methods have been investigated. In particular, intravenous injection is normally considered to have the advantage of avoiding the first-pass effect of hepatic metabolism, producing the highest bioavailability. When intravenous administration of edible tea flower nanoparticles was compared to oral administration, no difference was noted in terms of body weight and main pro-inflammatory cytokines levels. However, a sharp increase of complement C3 concentrations was detected, suggesting a slight immune reaction induced by these nanoparticles when they are administered intravenously (IV) (Chen et al., 2022). Other studies have suggested that IV administration of ginger derived exosome-like nanovesicles (GDELN) did not promote an immune reaction, though only body weight was examined (Li et al., 2018). The slight immune reaction induced by repetitive intravenous injection of EVs appears non-specific to plant EVs since a mild immune response has also been reported for human EVs (Saleh et al., 2019). The authors found that EVs purified from different sources could induce different responses. Therefore, this could also be the case for plant EVs, and more information needs to be collected before a conclusion could be drawn on intravenous injections of plant EVs. In parallel, one study has reported that intranasal administration of engineered grapefruit-derived nanovectors (GNVs) could slow down tumour brain progression in mice (Zhuang et al., 2016). This brings hope for the use of plant EVs as therapeutic tools in neurodegenerative diseases. It is noticeable that EV biodistribution changes with the administration method. While intravenous injection of mammalian and plant EVs results in the wide uptake by various organs (including spleen, liver, kidney, lung, heart, and brain) (Lai et al., 2014; Garaeva et al., 2021), the gut is more specifically targeted in oral administration of edible EVs (Ju et al., 2013; Mu et al., 2014; Zhang et al., 2016; Deng et al., 2017; Teng et al., 2018). In addition, plant EVs have been shown to penetrate a human skin model, which encourages their consideration for skin care treatments (Lee et al., 2020). Altogether, the data accumulated suggests that specific administration methods would have to be developed depending on the pathology targeted and that plant EVs present a lot of potential in therapeutic processes.

## 5 Conclusion

Extracellular vesicles (EVs) are associated with Unconventional protein secretion (UPS) routes. They are released in the extracellular space through mechanisms that are still poorly understood. The field of plant EVs is relatively new but is proving to have great prospects in biomedicine. The potential to produce pure plant EV subtypes such as exosomes through biopharming and be able to deliver therapeutic molecules is very appealing. Additional advantages include the engineering capability of *in vivo* cargo loading associated with low production costs and easy extraction procedures. Before validating plant EVs as putative drug delivery tools, further research investigating their toxicity and immunogenicity needs to be undertaken. In addition, a more robust composition and characterization of plant EVs is also essential in order to standardise production for good manufacturing practice (GMPs). Nevertheless, preliminary data seem very promising such as the efficient uptake of plant EVs by human cells, their expected low immunogenic character (associated with nutrition) and their positive effect on human health. As a consequence, using plant EVs as a drug delivery tool might represent a powerful future alternative to classical therapeutic systems.

## Abbreviations

EVs, extracellular vesicles; ER, endoplasmic reticulum; PM, plasma membrane; MVB, multivesicular body; TGN, trans-Golgi network; EE, Early endosome; ILVs, Intraluminal vesicles
